# The Role of 3D/4D Transperineal Ultrasound in Risk Stratification for Pelvic Organ Prolapse Recurrence: Native Tissue Versus Mesh Repair

**DOI:** 10.3390/jcm15124627

**Published:** 2026-06-14

**Authors:** José Antonio García-Mejido, María José Nuñez-Matas, Olaya Salas-Álvarez, Alejandro Crespo-Rodriguez, Ana Fernández-Palacín, José Antonio Sainz-Bueno

**Affiliations:** 1 Obstetrics and Gynecology Area, Department of Surgery, Faculty of Medicine, University of Seville, 41009 Seville, Spain; olaya.salas.sspa@juntadeandalucia.es (O.S.-Á.); jsainz@us.es (J.A.S.-B.); 2 Department of Obstetrics and Gynecology, Hospital Universitario Virgen de la Victoria, 29010 Málaga, Spain; mananemj@gmail.com (M.J.N.-M.); alexcrm97@gmail.com (A.C.-R.); 3 Biostatistics Unit, Department of Preventive Medicine and Public Health, University of Seville, 41009 Seville, Spain; afp@us.es

**Keywords:** pelvic organ prolapse, transperineal ultrasound, surgical mesh, native tissue repair, levator ani muscle, prolapse recurrence

## Abstract

**Background/Objectives**: Pelvic organ prolapse (POP) management requires precise patient selection for surgical techniques to balance clinical efficacy and safety. The primary aim of this study was to evaluate the role of preoperative 3D/4D transperineal ultrasound in the risk stratification of POP recurrence. We analyzed the impact of levator ani muscle (LAM) injuries, specifically avulsion and ballooning, as identified by ultrasound, on both anatomical and subjective success rates, comparing native tissue repair versus mesh-augmented surgery. **Methods**: A prospective, multicenter observational study was conducted over a five-year period, January 2021 to December 2024 (recruitment), with follow-up completed in December 2025, ensuring a minimum follow-up of 12 months for all participants. The cohort included 276 women scheduled for primary surgery for symptomatic POP stage ≥ 2. Prior to intervention (116 underwent native tissue repair and 160 received mesh), all patients underwent 3D/4D transperineal ultrasound for standardized volume acquisition. Using this preoperative functional imaging technique, we measured the hiatal area and diagnosed the presence of hiatal ballooning (≥25.0 cm^2^) or levator muscle avulsion. **Results**: Ultrasound assessment revealed significant differences in surgical success based on the diagnosed baseline site-specific defects. Hiatal ballooning was the sonographic finding that demonstrated the greatest impact on risk stratification. Among patients with preoperative ballooning, mesh use significantly reduced both subjective recurrence (5.7% vs. 21.4%, *p* = 0.001) and objective recurrence (21.4% vs. 35.7%, *p* = 0.040) compared to native tissue repair. Furthermore, in women without ultrasound-documented avulsion, mesh also decreased objective recurrence (17.9% vs. 33.0%, *p* = 0.024). Multivariate analysis, adjusted for age, BMI, menopausal status, and parity, confirmed that, after stratifying by these preoperative ultrasound findings, a native tissue approach remains the primary independent predictor of surgical failure (OR 1.752 for objective recurrence; *p* = 0.041). **Conclusions**: In conclusion, native tissue repair was identified as the primary independent predictor of surgical failure. While 3D/4D transperineal ultrasound helps identify high-risk phenotypes such as hiatal ballooning, these sonographic findings did not maintain independent significance in the multivariate model. Therefore, ultrasound should be considered a complementary tool for surgical planning rather than a definitive predictor of recurrence.

## 1. Introduction

Pelvic organ prolapse (POP) is a remarkably prevalent condition, affecting up to 60% of multiparous women. While native tissue repair has traditionally served as the standard surgical approach, this strategy is frequently compromised by notably high long-term anatomical failure rates. Research has indicated that recurrence risks following primary surgery can reach 30%, sometimes exceeding 50% when applying stringent anatomical criteria [1]. Such high failure rates are closely associated with underlying structural deficiencies [2].

To enhance anatomical durability and minimize recurrence, synthetic mesh reinforcements were introduced, successfully reducing the risk of objective recurrence by up to 50% [3,4]. However, the emergence of complications, including erosion and chronic pain, triggered significant safety warnings from regulatory bodies [5]. These issues are associated with exposure rates of approximately 12% and a heightened risk of reoperation [4,6,7]. Consequently, many countries have restricted their use, compelling surgeons to adopt a far more cautious approach to their practice [8]. Current international guidelines, such as those from the American College of Obstetricians and Gynecologists (ACOG) [7] and the International Urogynecological Association (IUGA), now recommend that transvaginal mesh be reserved for patients at particularly high risk of recurrence or for the surgical management of recurrent prolapse [7].

In this landscape, the primary challenge for modern urogynecology is the accurate identification and selection of candidates for each specific technique, striking a balance between anatomical efficacy and safety profiles [3,9,10]. Within this framework, 3D/4D transperineal ultrasound has emerged as a useful tool for the anatomical assessment of the pelvic floor, although its independent predictive value for surgical success remains to be fully established [10,11]. It has been demonstrated that surgical failure is not a random occurrence but is instead closely tied to pre-existing structural damage [10,12].

Levator ani muscle (LAM) avulsion has been shown to increase the risk of objective recurrence following anterior colporrhaphy by three to four times [9,11,13,14]. Nevertheless, recent meta-analyses suggest that its influence on symptomatic recurrence might be less decisive than previously thought [15,16,17]. Concurrently, LAM ballooning serves as a predictor of recurrence risk independently of avulsion [18,19].

Preoperative detection of these defects allows for a more individualized risk assessment, anticipating the potential failure of techniques that rely solely on the endopelvic fascia [20]. Although research such as that by [3] has shown that mesh significantly benefits patients with LAM avulsion and advanced POP, reducing recurrence risk by up to fourfold, further evidence is still required to understand how the combination of these structural injuries dictates the outcome of various surgical techniques.

We hypothesized that preoperative LAM damage (avulsion and ballooning) significantly elevates the risk of recurrence in native tissue repairs but is mitigated by mesh use. Therefore, this study aimed to evaluate the impact of these structural injuries on subjective and objective POP recurrence rates by comparing mesh repair versus native tissue repair.

## 2. Materials and Methods

### 2.1. Patient Inclusion

A prospective, multicenter observational study was designed and conducted. The primary recruitment period spanned from 1 January 2021 to 31 December 2024, across two public hospitals in Spain. Follow-up for the cohort concluded in December 2025, ensuring a minimum follow-up period of 12 months for all participants. The research protocol received approval from the Biomedical Research Ethics Committee of Andalusia (Ref: SICEIA 2026000105). This study was conducted in strict adherence to the principles of the Declaration of Helsinki (2013 revision), and all participants provided written informed consent prior to inclusion.

Throughout the study period, a consecutive cohort of 276 women scheduled for corrective POP surgery was recruited. The primary inclusion criterion was the presence of symptomatic POP, defined as Stage ≥ 2 in any compartment according to the Pelvic Organ Prolapse Quantification (POP-Q) system, with an indication for primary surgery following the failure or refusal of conservative management. Specifically, all included patients reported bothersome symptoms of a vaginal bulge with POP at or below the hymen. Exclusion criteria included any history of pelvic floor reconstructive surgery (for prolapse or incontinence) and the presence of known neurological pathology with potential impact on pelvic floor function.

### 2.2. Surgical Interventions

Patients were categorized into two study groups based on the primary surgical approach: native tissue repair or mesh-augmented repair. Allocation to each group was determined by the patient’s hospital of origin, reflecting the standardized protocol and routine surgical practice at each center. This assignment reflects routine clinical practice across the participating institutions; however, as a non-randomized observational study, it entails an inherent selection bias. While multivariate adjustments were employed to address baseline differences, this design-related factor should be considered when interpreting the comparative outcomes. The Hospital Universitario de Valme (Seville) provided the cohort of patients undergoing exclusive native tissue (autologous) repair; these interventions included anterior colporrhaphy, vaginal hysterectomy with McCall culdoplasty, the Manchester procedure, and posterior colporrhaphy. Conversely, the Hospital Universitario Virgen de la Victoria (Málaga) provided the cohort of patients undergoing mesh repair, which included approaches such as colposacropexy and bilateral sacrospinous suspension (BSC Mesh). To ensure technical consistency, all procedures were performed by the expert urogynecology surgical team corresponding to each institution. Concomitant anatomical defects (e.g., cervical elongation) were addressed during the same surgical time to ensure comprehensive anatomical correction.

### 2.3. Clinical Evaluation, Follow-Up, and Outcome Definitions

Baseline clinical evaluation was performed during the preoperative consultation following a standardized protocol for collecting demographic variables and obstetric history. POP staging was systematically performed in the dorsal lithotomy position using the POP-Q system during a maximum strain maneuver. The postoperative follow-up protocol was identical for both participating centers and was structured in two phases. At 3 months post-intervention, all patients were evaluated in person (both clinically and sonographically) to confirm initial anatomical success. Subsequently, a long-term follow-up was established via an annual consultation protocol, where patients were actively questioned regarding prolapse symptoms and underwent clinical evaluation. Subjective recurrence was defined as the reappearance of the vaginal bulge symptom reported by the patient at any point during follow-up. Objective recurrence was defined as the anatomical confirmation of Stage ≥ 2 prolapse (according to the POP-Q system) in any evaluated compartment during successive visits.

### 2.4. Ultrasound Examination

All examinations (each with over 10 years of experience in pelvic floor imaging) were performed by a single expert per center, blinded to clinical and surgical details. To ensure comparability and minimize inter-center bias, standardized protocols were followed using identical ultrasound technology and probes (Toshiba^®^ Aplio 700 and 500; PVT-675 MV, Canon Medical Systems Corporation, Otawara, Tochigi, Japan).

**Figure 1:**
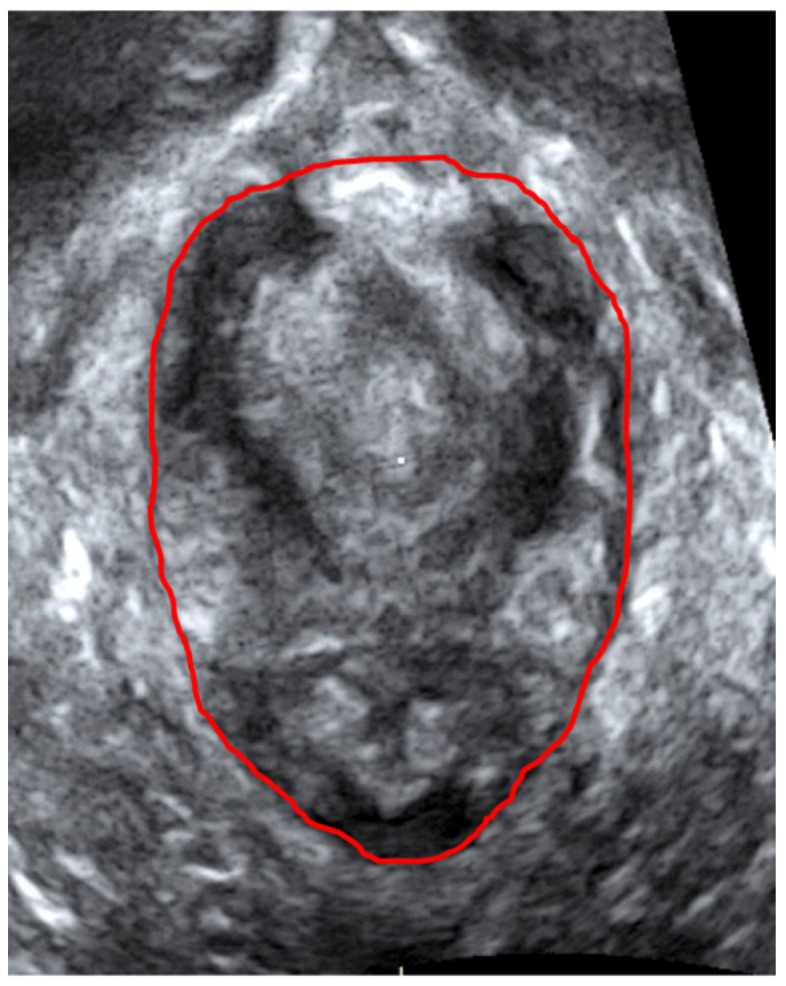
Hiatal ballooning was defined as an LHA ≥ 25.0 cm^2^ during maximum effort.

**Figure 2:**
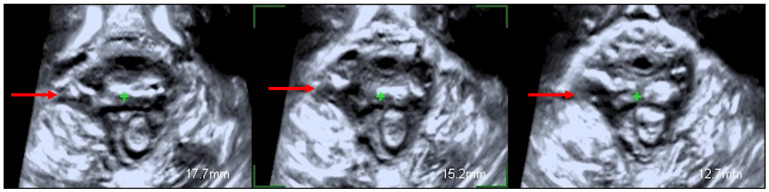
Avulsion was diagnosed upon the loss of normal muscle insertion at the pubis in the three central slices.

Patients were examined in the dorsal lithotomy position, with the probe placed on the perineum using minimal pressure. Standardized 3D volumes were acquired at rest, during contraction, and during a maximum, sustained Valsalva maneuver of at least 6 s. Analysis of the acquired volumes was performed directly on the ultrasound workstation by the same examiner who performed the capture at each center. The plane of minimal dimensions was identified, defined as the axial plane with the shortest distance between the posteroinferior border of the symphysis pubis and the anterior border of the levator ani muscle (LAM). In this plane, the levator hiatal area (LHA, in cm^2^) was measured, representing the space bounded by the levator ani muscle and the symphysis pubis. Hiatal ballooning was defined as an LHA ≥ 25.0 cm^2^ during maximum effort, reflecting an objective loss of structural integrity and an overdistension of the pelvic floor (Figure 1), according to the criteria established by Dietz and Simpson [21]. The diagnosis of LAM avulsion was evaluated during maximum contraction using tomographic ultrasound imaging in the plane of minimal dimensions, with 2.5 mm slice intervals. Avulsion was diagnosed upon the loss of normal muscle insertion at the pubis in the three central slices (Figure 2). In borderline cases, abnormal insertion was confirmed by measuring a levator–urethra gap > 2.5 cm.

### 2.5. Statistical Analysis

A complete-case analysis was performed. Cases with inconclusive ultrasound data were excluded from the corresponding analyses, and no imputation methods were applied due to the low proportion of missing data. Statistical analysis was conducted using the SPSS statistical package, version 31.0 (IBM Corp., Armonk, NY, USA). In the descriptive analysis, quantitative variables are expressed as mean ± standard deviation (SD) or median and interquartile range (IQR), depending on their distribution. Categorical variables are presented as absolute frequencies (n) and percentages (%). The normality of continuous variables was assessed using the Kolmogorov–Smirnov test. For bivariate analysis, the comparison of continuous variables between two independent groups was performed using Student’s *t*-test or the Mann–Whitney U test, as appropriate. The association between qualitative variables was analyzed using the chi-square test or Fisher’s exact test when expected frequencies were less than 5. To identify independent risk factors associated with surgical failure (subjective and objective recurrence) and to control for potential confounding factors (indication bias), a multivariate binary logistic regression model was constructed. Predictor variables that showed a statistically significant association in the univariate analysis (*p* < 0.10) or were considered clinically relevant (ballooning, parity, surgical technique) were included in the model. The results of the multivariate model are expressed as odds ratios (ORs) with their respective 95% confidence intervals (95% CIs). In all tests, a *p*-value < 0.05 was considered statistically significant. The number of events per variable was taken into account to minimize the risk of overfitting, particularly for the primary outcome of objective recurrence. The time to recurrence was analyzed as a secondary descriptive variable using mean and standard deviation to provide clinical context on failure patterns between surgical groups.

The sample size was calculated a priori based on the primary objective of the study: to compare the long-term objective recurrence rate between autologous tissue repair and mesh use in advanced prolapse. According to the recent literature based on comparative cohorts with similar characteristics [3], an anatomical failure rate (objective recurrence) of 46.0% was estimated for native tissue repair, and it was hypothesized that the use of prosthetic reinforcement would reduce this incidence to 20.2%. Accepting an alpha risk of 0.05 (two-tailed) and a beta risk of 0.20 (80% statistical power), it was determined that a minimum of 52 patients per group was required. To account for a potential 15% loss during the follow-up period, the minimum required sample size was set at approximately 62 patients per cohort. Since our final sample significantly exceeded this safety threshold (116 native tissue interventions and 160 mesh interventions), the analyzed cohort possesses high robustness and statistical power to detect significant clinical differences.

## 3. Results

During the recruitment period, a total of 276 patients met the clinical inclusion criteria. Within the overall cohort of 276 women, 116 (42.0%) underwent reconstructive surgery using autologous (native) tissue, while 160 (58.0%) were treated with mesh repair. Baseline demographic, clinical, and obstetric characteristics are detailed in Table 1. Baseline demographic and clinical characteristics showed several significant differences between the cohorts. Specifically, the mesh group was slightly older (60.9 vs. 59.2 years, *p* = 0.012) and presented a significantly more severe baseline anatomical profile, with a higher prevalence of cystocele, uterine prolapse, and rectocele, as well as a higher incidence of LAM injuries (ballooning and avulsion) (all *p* < 0.05).

To analyze the impact of sonographic findings on surgical success, the initial cohort (n = 276) was adjusted due to the exclusion of a small proportion of preoperative ultrasound images that were inconclusive for a definitive morphological diagnosis. Specifically, in the mesh repair group (n = 160), it was not possible to conclusively evaluate the presence of levator ani muscle (LAM) avulsion in 4 patients (final n analyzed for this variable = 156) or hiatal ballooning in 6 patients (final n analyzed = 154) due to artifacts during 3D/4D volume acquisition. The native tissue repair group (n = 116) maintained evaluable volumes for both parameters across the entire sample.

The assessment of transperineal ultrasound findings on surgical success rates revealed significant differences based on the baseline site-specific defects and the surgical technique employed. When analyzing the presence of LAM avulsion (Table 2), mesh repair demonstrated a statistically significant reduction in objective recurrence compared to native tissue in the subgroup of patients without documented avulsion (17.9% vs. 33.0%, *p* = 0.024). In contrast, for the subgroup of patients with avulsion, objective recurrence rates showed no statistically significant differences between the two surgical options (21.8% vs. 31.3%, *p* = 0.624). Descriptive analysis of the chronology of failures suggested that recurrences in the mesh group occurred earlier (340.1 ± 157.1 days) than in the native tissue group (724.1 ± 498.9 days). It must be emphasized that these temporal data are strictly descriptive and should be interpreted with caution, as the study was not designed or powered to analyze time to recurrence. However, in the group with avulsion, no statistically significant differences were observed in the time to recurrence between the two techniques.

The sonographic finding that demonstrated the greatest impact on risk stratification was hiatal ballooning (Table 3). In the critical subgroup of patients presenting with preoperative ballooning, mesh use drastically reduced both subjective recurrence (5.7% vs. 21.4%, *p* = 0.001) and objective anatomical recurrence (21.4% vs. 35.7%, *p* = 0.040) compared to native tissue repair. This finding highlights a clear protective effect of meshes in scenarios of severe hiatal site-specific compromise. However, it should be noted that the subgroup of patients without preoperative ballooning who received mesh repair was limited (n = 14); consequently, the results for this specific cohort should be interpreted with caution due to restricted statistical power.

Given the differences in baseline clinical severity between the study groups (indication bias inherent to mesh use in more complex prolapses), a multivariate binary logistic regression model was constructed to identify independent predictors of long-term surgical failure (Table 4). After adjusting for age, body mass index, menopausal status, and preoperative ultrasound findings, the choice of surgical technique was confirmed as the primary independent prognostic factor. Specifically, native tissue repair increased the risk of subjective recurrence more than fourfold (OR 4.575; 95% CI: 1.965–10.654, *p* < 0.001) and significantly increased the risk of objective recurrence (OR 1.752; 95% CI: 1.024–2.999, *p* = 0.041) compared to mesh use. Additionally, parity was identified as an independent risk factor for subjective recurrence (OR 1.809; 95% CI: 1.175–2.787, *p* = 0.007). In the overall multivariate model, neither avulsion nor ballooning maintained statistical significance as independent risk factors, confirming that the chosen surgical technique is the primary determinant of long-term success, regardless of baseline sonographic characteristics.

## 4. Discussion

The primary finding of this study is that native tissue repair constitutes the most significant independent risk factor for surgical failure in women with advanced POP. Following rigorous statistical adjustment, the use of autologous tissue increased the risk of subjective recurrence more than fourfold (OR 4.575) and significantly heightened the probability of objective recurrence (OR 1.752). This occurred despite the fact that the mesh-treated group presented with more severe baseline POP and a higher prevalence of cystocele. These data suggest a marked indication bias where, even when patients with the poorest anatomical prognosis were treated with prosthetic reinforcements, the outcomes were consistently superior to those achieved with traditional surgery.

The biological interpretation of these results is rooted in the biomechanics of the levator hiatus. Hiatal ballooning represents an overdistension of the hiatus and a loss of the structural integrity of the pelvic floor [21]. Our univariate data demonstrate that, in the presence of ballooning, mesh drastically reduces objective recurrence (21.4% vs. 35.7% in native tissue). It is biomechanically plausible that a repair based exclusively on the plication of the pre-existing, and already weakened, endopelvic fascia would fail when faced with an excessively distensible hiatus [20]. As suggested in the literature, an increased hiatal area implies that any supporting structure will be subjected to greater mechanical stress, leading to the elongation or rupture of autologous tissue [22].

Our results expand and refine the paradigm established by Wong et al. [3]. In their cohort of Asian women, Wong et al. [3] identified LAM avulsion as the critical factor justifying mesh benefit, reporting a significant reduction in recurrence following its use in patients with LAM avulsion. However, in our population, the determining sonographic factor that benefits from mesh protection is ballooning. Notably, in our multivariate analysis, neither avulsion nor ballooning maintained statistical significance independently. This suggests that a major factor associated with surgical failure is not the anatomical injury *per se*, but rather the decision to attempt a repair using the patient’s own damaged tissues. This finding aligns with the report by Liu et al. [22], underscoring that hiatal distensibility is a critical prognostic marker in surgical planning. In this sense, our findings agree with the biomechanical evidence provided by Handa et al. [23], who demonstrated that increased hiatal area and loss of muscle strength mediate up to 61% of the pathophysiological association between avulsion and POP. This reinforces our primary thesis: hiatal ballooning and, fundamentally, the chosen surgical technique, appear to be key factors influencing long-term anatomical success.

A novel clinical finding of our study is the marked difference in the chronology of surgical failure. Our analysis showed that in patients without avulsion, native tissue repair tends to fail late (with a mean exceeding two years post-intervention), whereas recurrence in the mesh group, when it occurs, happens early (before the first year). This temporal dichotomy, though purely descriptive in our cohort, has important pathophysiological and technical implications. The late failure of autologous tissue is congruent with the biomechanics of the endopelvic fascia, which undergoes a gradual process of elongation and structural degradation when subjected to sustained mechanical stress over the years [20,22]. Conversely, the early failure of meshes suggests that recurrence in these cases is not due to long-term tissue deterioration but may be closely linked to immediate technical factors, such as integration failure or sub-optimal anchoring of the mesh fixation arms during the initial healing process. This phenomenon highlights the need for a meticulous surgical technique when securing implants to avoid early failures.

From a clinical perspective, our data suggest that 3D/4D ultrasound provides valuable anatomical information; however, it should not be considered an independent predictor of failure. The multivariate model clearly indicates that the structural weakness of the patient’s own tissue (native tissue repair) is the factor that truly drives recurrence. While magnetic resonance imaging (MRI) remains a highly accurate diagnostic modality for pelvic floor assessment, 3D/4D transperineal ultrasound provides a more accessible and dynamic alternative, allowing for functional imaging during real-time effort. Identifying a high-risk site-specific phenotype, characterized by ballooning, allows clinicians to anticipate the failure of biological techniques. Identifying high-risk site-specific phenotypes is crucial given current mesh controversies. This model provides a framework to limit prosthetic exposure to high-risk cases where anatomical durability outweighs potential risks, such as mesh erosion or chronic pain, aligning with safety recommendations for targeted mesh use. Furthermore, surgical planning should be integrated into a multimodal approach. Complementary conservative strategies, such as pelvic floor rehabilitation and local estrogen therapy, are essential for improving vaginal tissue quality and support [24]. While previous meta-analyses have encountered difficulties in confirming avulsion as a definitive risk factor after adjusting for confounding variables [4], our study clarifies that the chosen surgical technique is the primary mediator of success. Parity was also confirmed as an independent risk factor for subjective recurrence (OR 1.809), reinforcing the idea that accumulated damage to support structures limits the durability of traditional repairs [25].

### Strengths and Limitations

Regarding methodological considerations, this work possesses notable strengths. Its multicenter design significantly increases the external validity of the results, demonstrating reproducibility across different clinical settings. Furthermore, the analyzed cohort (n = 276) represents one of the largest samples in the literature, combining 3D/4D ultrasound, surgical intervention, and long-term follow-up, the latter being essential for evaluating the true durability of pelvic compartment repairs. Additionally, the strict exclusion of inconclusive ultrasound images and the application of a multivariate statistical model ensure high analytical robustness.

Nonetheless, we must acknowledge the limitations inherent in its observational and non-randomized nature. Fundamentally, an indication bias exists because the choice of reconstructive technique was not randomized but depended on the evaluation of the multicompartmental profile, the severity of the defect in each patient, and the specific surgical protocols of each participating center. This center-dependent allocation introduces a potential confounding factor, as differences in outcomes could be influenced not only by baseline patient severity but also by institution-specific variations in surgical execution or perioperative care. In real-world clinical practice, mesh use was prioritized in cases with more complex anatomical defects or more evident risk factors for failure. While this bias was addressed through logistic regression analysis to isolate the effect of the surgical technique from baseline severity, our findings should be interpreted with caution. In future studies, the implementation of more advanced statistical methods, such as propensity score matching or further stratified analyses, would be beneficial to further minimize indication bias and strengthen the comparative validity of the results. Furthermore, it must be acknowledged that the outcomes observed in this study were achieved within specialized urogynecological units, where both the surgical interventions and the ultrasound assessments were performed by senior experts. This high level of specialization may limit the immediate generalizability of our results to general gynecology settings. The implementation of a personalized surgical model based on functional imaging requires a specific learning curve and technical proficiency in 3D/4D transperineal ultrasound, which should be considered when translating these findings to non-specialized centers. Consequently, specialized training in pelvic floor imaging is essential for the effective clinical adoption of this risk-stratification tool. Additionally, it should be noted that while this study provides robust evidence regarding anatomical and subjective recurrence, specific mesh-related complications such as erosion or chronic pain were not systematically recorded as primary outcomes. This represents a limitation for a comprehensive risk–benefit analysis, and future studies should integrate systematic safety monitoring alongside functional imaging to provide a more complete clinical perspective. Additionally, crucially, the absence of systematic data on mesh-related complications, such as erosion or chronic pain, precludes a definitive recommendation for mesh use. A complete risk–benefit analysis is necessary before prioritizing prosthetic reinforcement over native tissue repair, as current international safety warnings emphasize a cautious approach. Finally, although the POP-Q system was used as the standardized clinical tool for anatomical assessment, given its widespread international acceptance and comparability, we acknowledge that its clinical precision may be subject to inherent variability compared to other emerging measurement methods.

## 5. Conclusions

In conclusion, the choice of surgical technique (native tissue repair) is the main independent risk factor for POP recurrence. Although 3D/4D transperineal ultrasound allows for the identification of phenotypes with hiatal ballooning, these findings do not independently predict failure. Ultrasound remains a useful complementary tool for specialized surgical planning, but further randomized studies including complication rates are required to define its definitive role in clinical practice.

## Figures and Tables

**Table 1:** General clinical characteristics of the patients.

	All (n:276)	Native Tissue Repair (n:116)	Mesh Repair (n:160)	*p*
Age (years)	60.2 ± 9.2	59.2 ± 9.7	60.9 ± 8.8	0.012
Age at menopause (years)	49.8 ± 4.4	50.3 ± 4.0	49.3 ± 4.7	0.098
Weight (Kg)	69.9 ± 11.5	71.2 ± 12.0	68.9 ± 10.9	0.199
Height (m)	1.6 ± 0.1	1.6 ± 0.07	1.6 ± 0.06	0.573
BMI	27.1 ± 4.6	27.6 ± 4.7	26.7 ± 4.5	0.209
Obstetric history				
Parity	2.3 ± 1.1	2.4 ± 1.2	2.3 ± 1.0	0.542
Cesarean sections	0.1 ± 0.4	0.2 ± 0.5	0.08 ± 0.3	0.051
Cystocele	250 (90.6%)	97 (83.6%)	153 (95.6%)	0.004
Stage I	9 (3.3%)	3 (2.6%)	6 (3.8%)	0.003
Stage II	30 (10.87%)	18 (15.5%)	12 (7.5%)	
Stage III	198 (71.7%)	76 (65.5%)	122 (76.3%)	
Stage IV	13 (4.7%)	0 (0.0%)	13 (8.1%)	
Uterine prolapse	177 (64.1%)	43 (37.1%)	134 (83.8%)	<0.001
Stage I	19 (6.9%)	6 (5.2%)	13 (8.1%)	0.405
Stage II	51 (18.5%)	9 (7.8%)	42 (26.3%)	
Stage III	94 (34.1%)	26 (22.4%)	68 (42.5%)	
Stage IV	13 (4.7%)	2 (1.7%)	11 (6.9%)	
Cervical elongation	59 (21.4%)	52 (44.8%)	7 (4.4%)	<0.001
Stage I	4 (1.4%)	4 (3.4%)	0 (0%)	0.100
Stage II	16 (5.8%)	15 (12.9%)	1 (0.6%)	
Stage III	38 (13.8%)	33 (28.4%)	5 (3.1%)	
Stage IV	1 (0.4%)	0 (0%)	1 (0.6%)	
Rectocele	170 (61.6%)	42 (36.2%)	128 (80%)	<0.001
Stage I	47 (17.0%)	17 (14.7%)	30 (18.8%)	0.059
Stage II	94 (34.1%)	17 (14.7%)	77 (48.1%)	
Stage III	29 (10.5%)	8 (6.9%)	21 (13.1%)	
Enterocele	14 (5.1%)	9 (7.8%)	5 (3.1%)	0.157
Stage I	3 (1.1%)	2 (1.7%)	1 (0.6%)	0.237
Stage II	4 (1.4%)	4 (3.4%)	0 (0%)	
Stage III	7 (2.5%)	3 (2.6%)	4 (2.5%)	
LHA at Valsalva	33.2 ± 8.3	28.9 ± 8.2	36.7 ± 6.6	<0.001
Ballooning	210 (76.1%)	70 (60.3%)	140 (87.5%)	<0.001
Avulsion	94 (34.1%)	16 (13.8%)	78 (48.8%)	<0.001

Values are presented as mean ± standard deviation or n (%). BMI: body mass index; LHA: levator hiatal area.

**Table 2:** Analysis of recurrences based on the presence of levator ani muscle avulsion and surgical approach.

	With Avulsion & Native Tissue (n:16)	Without Avulsion & Native Tissue (n:100)	With Avulsion & Mesh (n:78)	Without Avulsion & Mesh (n:78)	*p*1	*p*2	*p*3	*p*4
Subjective recurrence	3 (18.8%)	20 (20.0%)	3 (3.8%)	5 (6.4%)	0.825	0.717	0.097	0.018
Objective recurrence	5 (31.3%)	33 (33.0%)	17 (21.8%)	14 (17.9%)	0.882	0.688	0.624	0.037
Time to recurrence (days)	694.8 ± 555.1	724.1 ± 498.9	512.3 ± 425.5	340.1 ± 157.1				
Cystocele recurrence	5 (31.3%)	27 (27%)	14 (17.9%)	13 (16.7%)	0.959	1.000	0.387	0.145
Uterine prolapse recurrence	0 (0%)	5 (5%)	3 (3.8%)	1 (1.3%)	0.802	0.613	0.987	0.345
Cervical elongation recurrence	0 (0%)	0 (0.0%)	1 (1.3%)	1 (1.3%)	---	0.477	0.378	0.901
Rectocele recurrence	0 (0%)	15 (15.0%)	3 (3.8%)	0 (0%)	0.210	0.244	0.987	0.122
Enterocele recurrence	0 (0%)	0 (0%)	0 (0%)	0 (0%)	---	---	---	---

The analyzed sample size varies slightly from the total cohort (N = 276) due to the exclusion of inconclusive preoperative ultrasound images. *p*1: with avulsion & native tissue vs. without avulsion & native tissue. *p*2: with avulsion & mesh vs. without avulsion & mesh. *p*3: with avulsion & native tissue vs. with avulsion & mesh. *p*4: without avulsion & native tissue vs. without avulsion & mesh.

**Table 3:** Analysis of recurrences based on the presence of levator ani muscle ballooning and surgical approach.

	With Ballooning & Native Tissue (n:70)	Without Ballooning & Native Tissue (n:46)	With Ballooning & Mesh (n:140)	Without Ballooning & Mesh (n:14)	*p*1	*p*2	*p*3	*p*4
Subjective recurrence	15 (21.4%)	8 (17.4%)	8 (5.7%)	0 (0.0%)	0.768	0.774	0.001	0.219
Objective recurrence	25 (35.7%)	13 (28.3%)	30 (21.4%)	1 (7.1%)	0.526	0.357	0.040	0.202
Time to recurrence (days)	750.2 ± 500.7	662.7 ± 509.9	453.9 ± 343.2					
Cystocele recurrence	22 (31.4%)	10 (21.7%)	27 (19.3%)	0 (0.0%)	0.352	0.149	0.074	0.133
Uterine prolapse recurrence	3 (4.3%)	2 (4.3%)	4 (2.9%)	0 (0.0%)	---	---	---	---
Cervical elongation recurrence	2 (2.9%)	0 (0.0%)	1(0.7%)	1 (7.1%)	---	0.431	0.723	0.525
Rectocele recurrence	10 (14.3%)	5 (10.9%)	3 (2.1%)	0 (0%)	0.799	0.645	0.002	0.462
Enterocele recurrence	0 (0.0%)	0 (0.0%)	0 (0.0%)	0 (0%)	---	---	---	---

The analyzed sample size varies slightly from the total cohort (N = 276) due to the exclusion of inconclusive preoperative ultrasound images. *p*1: with ballooning & native tissue vs. without ballooning & native tissue. *p*2: with ballooning & mesh vs. without ballooning & mesh. *p*3: with ballooning & native tissue vs. with ballooning & mesh. *p*4: without ballooning & native tissue vs. without ballooning & mesh.

**Table 4:** Univariate and multivariate logistic regression analyses of independent risk factors for long-term subjective and objective surgical recurrence.

	Subjective Recurrence				Objective Recurrence			
	Univariate Analysis		Multivariate Analysis		Univariate Analysis		Multivariate Analysis	
	OR (95% CI)	*p*	OR (95% CI)	*p*	OR (95% CI)	*p*	OR (95% CI)	*p*
Age (years)	1.042 (1.002; 1.084)	0.041			1.020 (0.992; 1.049)	0.160		
Menopause	1.691 (0.736; 3.889)	0.216			1.396 (0.753; 2.589)	0.290		
Age at menopause	0.951 (0.853; 1.060)	0.362			0.961 (0.895; 1.033)	0.281		
BMI	1.007 (0.929; 1.091)	0.866			0.942 (0.889; 0.998)	0.044		
Parity	1.768 (1.158; 2.701)	0.008	1.809 (1.175; 2.787)	0.007	1.021 (0.810; 1.289)	0.858		
Cesarean sections	0.860 (0.350; 2.111)	0.741			1.037 (0.518; 2.097)	0.917		
LHA at rest	1.015 (0.933; 1.103)	0.735			1.027 (0.956; 1.103)	0.467		
LHA at Valsalva	1.019 (0.975; 1.066)	0.404			0.993 (0.960; 1.026)	0.669		
Ballooning	1.251 (0.529; 2.959)	0.610			0.858 (0.438; 0.681)	0.655		
Avulsion	2.397 (0.947; 6.067)	0.065			1.174 (0.656; 2.102)	0.589		
Native tissue repair	5.315 (2.315; 12.497)	<0.001	4.575 (1.965;10.654)	<0.001	1.786 (1.057; 3.020)	0.030	1.752 (1.024; 2.999)	0.041

## Data Availability

The dataset is available upon request from the authors.

## Abbreviations

BMI	Body Mass Index
BSC	Bilateral Sacrospinous
CI	Confidence Interval
IQR	Interquartile Range
LAM	Levator Ani Muscle
LHA	Levator Hiatus Area
POP	Pelvic Organ Prolapse
POP-Q	Pelvic Organ Prolapse Quantification System
SD	Standard Deviation
